# Crystal ball – 2017

**DOI:** 10.1111/1751-7915.12599

**Published:** 2017-01-27

Every 2 years, to kick off the New Year, Microbial Biotechnology publishes the Crystal Ball feature, in which leading researchers in the field of applied microbiology speculate on the technical and conceptual developments that will drive innovative research, open new vistas over the next few years and change current paradigms. The basic idea is that each contributor identifies such a development, describes its driver and analyses its anticipated impact on the field. The intention is to stimulate discussion, so caution is not *de rigueur*: wild (but founded) speculation is the norm. The feature is extremely popular and one of the most highly visited part of the MBT website. Moreover, the articles are freely downloadable, so enjoy a wide readership.

This year's diverse group of luminaries have shared with us an exciting spectrum of personal visions on how the future can look: enjoy, discuss and chase the future now!

**Antoine, Auxi, Juan Luis, Ken, Siegfried and Willem**

